# Patient complexity, depression, and quality of life in patients with epilepsy at an epilepsy center in Japan

**DOI:** 10.1002/epi4.12614

**Published:** 2022-05-31

**Authors:** Yasuiro Kishi, Ichiro Takumi, Hitoshi Yamamoto, Takako Ishimaru, Steven Thurber

**Affiliations:** ^1^ Department of Psychiatry Nippon Medical School Musashikosugi Hospital Kawasaki Japan; ^2^ Department of Neurosurgery St. Marianna University Kawasaki Japan; ^3^ Epilepsy Center St. Marianna University Kawasaki Japan; ^4^ Department of Pediatrics St. Marianna University Kawasaki Japan; ^5^ Department of Psychiatry and Behavioral Sciences University of Minnesota Minneapolis Minnesota USA

**Keywords:** care complexity, depression, epilepsy, epilepsy center, health‐related quality of life

## Abstract

**Objective:**

The relationship between care complexity and quality of life among patients with epilepsy has not been assessed, especially in Japan. The aim of this study is to test the hypothesis that care complexity is associated with health‐related quality of life (HRQOL) and mood disturbance.

**Method:**

This was an observational cross‐sectional study. The study included a consecutive series of 49 patients who newly visited an epilepsy center. Study participants were administered standardized quantitative measures of HRQOL, case complexity, and depression.

**Results:**

Patient complexity predicted lower HRQL scores. Data on the social and psychological complexity domains predicted patient HRQOL findings more accurately than data from the biological domain of the case complexity scale. Seizure frequency was unrelated to HRQOL findings in this study. Additionally, depression scores were also associated with lower HRQOL.

**Significance:**

A patient complexity assessment, including psychological and social domains, may be one of the key tools in epilepsy treatment settings. Further studies using larger random selection from patients with epilepsy are necessary to generalize the findings to patients in other epilepsy programs.

**Key Points**

- Care complexity predicted lower HRQL scores.
- Social and psychological complexity predicted patient HRQOL more accurately than biological complexity.
- Seizure frequency was unrelated to HRQOL findings in this study.
- Depression was associated with lower HRQOL.
- Complexity assessment may be one of the key tools in epilepsy treatment settings.

## 1 INTRODUCTION

Quality of life (QOL) is an important outcome measure in people living with epilepsy. In fact, QOL measures have become more important in the determinant of the health status of patients with chronic illness, including epilepsy, than measures of severity of the disease alone.^1^ Epilepsy is a complex neurological disease that requires medical, psychological, and social management. Further, patients with epilepsy need to complete many transitions throughout the health‐care system. Integrated health care has been proposed to deal with case and care complexity in many chronic disorders.^2^, ^3^, ^4^, ^5^, ^6^, ^7^ However, the relationship between care complexity and quality of life in patients with epilepsy has not been assessed, especially in Japan.

In an earlier study, depression, assessed by the PHQ‐9, was found in 29.3% of patients presenting to an epilepsy clinic.^8^ In a multicenter study evaluating pharmaco‐resistant patients with epilepsy, depressive symptoms were more important determinants of health‐related quality of life than seizures themselves.^9^ Other international studies, including Japanese studies, have also reported that depression is one of the stronger predictors of poor quality of life in patients with epilepsy.^10^, ^11^, ^12^, ^13^, ^14^, ^15^, ^16^, ^17^

The aim of this study was to test the hypothesis that care complexity is associated with health‐related quality of life and mood disturbance, with newly visited phase patients in an epilepsy center as research participants.

## 2 METHODS

### 2.1 Patients and study design

This was an observational cross‐sectional study. Eligible participants were 20 or older. The patients who had a diagnosis of moderate to severe level of dementia or did not have ability to comprehend these study procedures were not eligible in this study. During the study period (from October 1, 2019, to December 31, 2020), 221 patients were new patients of the epilepsy center at the St.Marianna University hospital. After assessment of the 221 patients, 70 patients were eligible for this study. Among the 70 patients, the investigation included a consecutive series of 49 patients who agreed to participate in the study. The hospital is a 1175‐bed hospital located in Kawasaki city, the ninth most populated city in Japan and one of the main cities forming the Greater Tokyo Area. The hospital provides tertiary care to the surrounding community. The epilepsy center was founded in 2017 to provide interdisciplinary comprehensive care services to patients with epilepsy. The study was approved by the ethics committee of St. Marianna University hospital.

### 2.2 Procedures

Data collected from patients included demographic characteristics, educational level, marital and work status, medical comorbidities, details on epilepsy history (including seizure types and seizure frequency per month), numbers of anti‐seizure medications, subjective adverse effects of anti‐seizure medications, presence of definite or suspected psychological nonepileptic seizures (PNES), history of epileptic surgery, and treatment of vagal nerve stimulation (VNS).

Study participants were assessed using the following standardized scales on the initial and/or second visit:

#### 2.2.1 QOLIE‐31 (Quality of Life in Epilepsy Inventory‐31)

Health‐related quality of life (HRQOL) was measured by the validated Japanese version of the QOLIE‐31, a widely used epilepsy‐specific self‐administered measure.^18^ The 31‐item questionnaire contains seven subscales (emotional well‐being, social function, energy/fatigue, cognitive function, seizure worry, medication effects, and overall quality of life) and one question about overall health status.^19^ Raw scores are converted to “0–100” range scores, with higher scores indicating better HRQOL.

#### 2.2.2 INTERMED

The INTERMED has been developed as a method for assessing case complexity and resulting care needs in order to foster multidisciplinary and integrated treatment.^20^, ^21^ Following information gathering and interviews, the trained research assistants completed the INTERMED Japanese version.^22^ The INTERMED^20^, ^21^ is an observer‐rated instrument that classifies information from a structured medical history‐taking encounter into four domains: biological, psychological, social, and health system. The domains are assessed in the context of time (historical and current state) and vulnerability. The information on 20 predictive risk variables that are scored on a scale from 0 (no problem) to 3 (immediate change is needed). Domain scores are obtained by adding scores of the five variables for each domain. The domain scores and the total scores of the INTERMED, therefore, may range from 0 to 15 and 0 to 60, respectively, with higher scores indicating higher complexity. The cut‐off score to identify the need for integrated care is 21 or above.

#### 2.2.3 PHQ‐9 (Patient Health Questionnaire‐9)

Depression was assessed by a validated Japanese version of PHQ‐9.^23^ The PHQ‐9 is a nine‐item questionnaire designed to screen for depression in primary care and other medical settings.^24^, ^25^, ^26^ The standard cut‐off score for screening to identify possible major depression is 10 or above.

### 2.3 Statistics

For comparison of parametric data in two groups, appropriate two‐sample *t* tests were performed based on equal or unequal variances by the Levene's test. To compare more than two groups, one‐way analysis of variance was used. To document correlations between continuous variables, the Pearson product–moment correlation coefficient was computed. Final associated factors were identified by an ANCOVA (analysis of covariance), including variables with a *P*‐value <.10 in the univariate analysis. All *P*‐values were two‐tailed. All data analyses were conducted using Statistical Product and Service Solutions (SPSS) statistical software 25.0.

There were three individuals who deviated markedly from the core distribution in terms of elevated numbers of seizures per month. Similarly, three patients evinced low‐frequency episodes that deviated from the main distribution. The Winsorization procedure for outlier adjustments was employed.^27^ The three high‐frequency outliers scores of 30, 90, and 2000 were replaced by the preceding “good” value of 14 in the distribution. This was balanced by substituting the “good” distribution value of 0.10 for low episodes scores of 0.00, 0.02, and 0.08.

## 3 RESULTS

### 3.1 Patient characteristics

Table 1 shows the patient characteristics. Older patients obtained significantly lower QOLIE‐31 total scores (*r* = −.43, *P* = .002). Male patients also showed significantly lower QOLIE‐31 health quality of life total scores than females (t = 2.4, df; 46.7, *P* = .021). Patients with medical comorbidities had a trend for lower QOLIE‐31 total scores than those without (t = 1.9, df; 47, *P* = .060). There was a trend for lower QOLIE‐31 total scores for the patients with subjective adverse effects of anti‐seizure medications compared with those without (t = 1.8, df; 47, *P* = .080). Married patients had a trend for lower QOLIE‐31 total scores than unmarried patients (t = 2.0, df; 46, *P* = .050). Other variables, including numbers of seizure attacks per month, were not correlated with QOLIE‐31 total scores.

**TABLE 1:** Patient characteristics

Sex, male, n (%)	22 (44.9)
Age (years), Mean (SD)	34.5 (10.7)
Education (years), Mean (SD)	14.4 (2.0)
Marital, married, n (%)	14 (28.6)
Employment, unemployed, n (%)	13 (26.5)
Medical comorbidities, presence, n (%)	8 (16.3)
Seizure types, n (%)	
Focal	38 (77.6)
Generalized	6 (12.2)
Unknown	5 (10.2)
Disease duration (years), Mean (SD)	16.4 (13.1)
Seizures per month (numbers), Mean (SD)	4.6 (6.2)
Anti‐seizure medications (numbers), Mean (SD)	2.4 (1.1)
Presence of subjective adverse effects, n (%)	24 (49.0)
Presence of PNES (possible/definite), n (%)	10 (20.4)
History of epilepsy surgery, n (%)	4 (8.2)
VNS treatment, n (%)	4 (8.2)

Abbreviations: PNES, Psychological non‐epileptic seizures; SD, standard deviation; VNS, vagal nerve stimulation.

### 3.2 QOLIE‐31, INTERMED, and PHQ‐9

Table 2 shows data from rating scales including the QOLIE‐31, the INTERMED, and the PHQ‐9. High complexity patients (INTEMED scores ≧21) had significantly lower QOLIE‐31 total scores than the below cut‐off complexity patients (t = 3.8, df; 47, *P* < .001). The patients with depression (24.5% of the patients) (PHQ total scores≧10) had significantly lower QOLIE‐31 total scores than those without depression (t = 5.9, df; 27.0, *P* < .001). Among the 12 depressed patients, only three patients had received antidepressants‐treatment. High complexity patients (INTEMED scores ≧21) had significantly higher PHQ total scores (t = −3.7, df; 47, *P* < .001). There was a trend for higher PHQ total scores for the patients with subjective adverse effects of anti‐seizure medications compared with those without (t = 1.8, df; 47, *P* = .074).

**TABLE 2:** INTERMED, QOLIE‐31‐P､PHQ‐9scores

QOLIE‐31‐P	
Total scores, Mean (SD)	60.1 (17.4)
Worry, Mean (SD)	36.6 (26.4)
QOL, Mean (SD)	52.1 (17.3)
Emotional, Mean (SD)	63.1 (19.3)
Energy, Mean (SD)	59.6 (19.2)
Cognitive, Mean (SD)	69.8 (25.3)
Medication effects, Mean (SD)	58.6 (30.6)
Social function, Mean (SD)	60.1 (28.5)
INTERMED	
Total scores, Mean (SD)	15.0 (7.9)
≧21、n (%)	11 (22.4)
Biological, Mean (SD)	5.9 (3.1)
Psychological, Mean (SD)	4.4 (2.8)
Social, Mean (SD)	2.9 (2.4)
Health System, Mean (SD)	1.8 (2.0)
PHQ‐9	
Total scores, Mean (SD)	7.6 (5.2)
≧10、n (%)	12 (24.5)

*Note*: QOLIE‐31 = Quality of Life in Epilepsy Inventory‐31; Possible range is 0 to 100, with higher scores indicating better quality of life.

INTERMED; Possible total scores range is 0 to 60 and the domain scores range is 0 to 15, with higher scores indicating higher complexity. The cut‐off score (“complex” patients) is 21 or above.

PHQ‐9 = Patient Health Questionnaire; Possible range is 0 to 27, with higher scores indicating more depressed. The standard cut‐off score for screening to identify possible major depression is 10 or above.

Abbreviation: SD, standard deviation.

As depicted in Table 3, the total INTERMED complexity score correlated significantly with depression as measured by PHQ‐9. The total INTERMED complexity score correlated inversely and significantly with the HRQOL measure (QOLIE‐31). In addition, all INTERMED subscales evinced significant negative relationships with the HRQOL measure (QOLIE‐31).

**TABLE 3:** INTERMED correlations with PHQ‐9 depression and QOLIE‐31, health‐related quality of life (HRQOL)

Variables	*r*	*P*
Total/PHQ‐9	.579	.001
Total/QOLIE‐31	−.700	.001
Biological/QOLIE‐31	−.540	.001
Psychological/QOLIE‐31	−.581	.001
Social/QOLIE‐31	−.620	.001
Health System/QOLIE‐31	−.389	.006

Abbreviations: PHQ‐9, Patient Health Questionnaire; QOLIE‐31, Quality of Life in Epilepsy Inventory‐31.

Patient complexity (INTERMED) and depression (PHQ‐9) data were regressed against scores on the HRQOL assessment (QOLIE‐31). The obtained *R* was .832 (*R* ^2^ = .692; F = 12.8, df = 7; 40 *P* < .001). The relative contributions (standardized beta weights) for the predictor variables are presented in Table 4.

**TABLE 4:** Multiple regression modeling patient complexity and depression in relation to QOLIE‐31, health related quality of life (HRQOL)

Predictor	Beta weight (β)	*P*
INTERMED (Patient Complexity)	−.299	.015
PHQ‐9 (Depression)	−.510	.001

Abbreviations: PHQ‐9, Patient Health Questionnaire; QOLIE‐31, Quality of Life in Epilepsy Inventory‐31.

Following stepwise regression of the INTERMED subscale domain scores on PHQ‐9 depression, only the “psychological” variable remained (see Table 5).

**TABLE 5:** Stepwise regression of INTERMED psychological subscale in relation to PHQ‐9 depression

Predictor	Beta weight (β)	*P*
Psychological	.617	.001
	*R* = .617, F = 28.9, df = 1;47 *P* < .001	

Abbreviation: PHQ‐9, Patient Health Questionnaire.

Stepwise regression of the four INTERMED domain scale scores in relation to HRQOL (QOLIE‐31), indicated that the “social” and “psychological” subscales obtained significant standardized weights. These are displayed in Table 6.

**TABLE 6:** Stepwise regression of INTERMED subscales in relation to COLIE‐31, health related quality of life (HRQOL)

Predictor	Beta weight (β)	*P*
Social	−.454	.001
Psychological	−.385	.001
	*R* = .771, F = 23.485, df = 7/40 *P* < .001	

Abbreviations: PHQ‐9, Patient Health Questionnaire; QOLIE‐31, Quality of Life in Epilepsy Inventory‐31.

## 4 DISCUSSION

Our findings showed that care complexity, assessed by the INTERMED, predicted poorer health‐related quality of life (HRQOL). Depression also predicted poorer HRQOL, consistent with other studies.^9^, ^10^, ^11^, ^12^, ^13^, ^14^, ^15^, ^16^, ^17^ Our results showed that 24% of the study participants were classified as clinical depression, which consistent with a meta‐analysis estimated that prevalence for major depression disorder among patients with epilepsy was 22%.^28^ In an availability sample of patients with epilepsy, two patient complexity subscales, social and psychological, evinced substantial relationships with patient HRQOL (Table 3). As displayed in Table 6, the combination of these two complexity domain variables accounted for almost 60% of the variance in the patient reports of their HRQOL (*R* = .771; *R* ^2^ = .594). Also as presented in Table 6, social difficulties obtained the highest negative beta weight in the multiple regression equation. This indicates that when social complexity problems increase by one standard deviation, the patient’s HRQOL will decrease by that Beta value. And as psychological problems increase by the standard deviation unit, there will be an additional reduction in the HRQOL equal to Beta for participants in our sample. The results suggest that complexity assessments may be essential elements for revealing non‐clinical areas that likely need clinical attention including ineffectively treated epilepsy, social isolation, an undiagnosed mental illness, poor relations with health‐care workers, and so on. In addition, only 25% of the depressed patients received antidepressants treatment in this study, which suggests many depressed patients may have not been adequately treated.

Before further discussion, it is important to acknowledge the methodological limitations of the study. First, this study represents patients in only one epilepsy center. The question remains regarding the external validity, or degree of generalization of such findings. Without random selection from the target population of adult patients with epilepsy, generalization is of course not assured. Nonetheless, the current results are at least consciously raising with respect to the importance of monitoring areas of patient complexity; in particular, interpersonal deficits and social support among patients with epilepsy. Second, we assessed adverse effects of anti‐seizure medications using a dichotomized (yes/no) scale lacking previous validation; and several studies have shown that anti‐seizure medications side effects and toxicity negatively affect HRQOL in patients with epilepsy using validated assessment scales. For instance, scores on the adverse events profile (AEP) were strongly correlated with HRQOL.^9^, ^29^ Our study may have underestimated the impact of anti‐seizure medications side effects.

Psychological (psychiatric) and biological factors can interact and contribute to complex patients' presentations and outcomes. For example, psychiatric comorbidities are negatively associated with postoperative seizure‐freedom rates after temporal lobe surgery.^30^ Further, several studies have suggested that comorbid depression^31^, ^32^, ^33^, ^34^ is a risk factor for seizures. Treating comorbid depression might affect the degree of seizure control. An analysis of the US Food and Drug Administration data on clinical trials with 75 873 participants showed that those given antidepressants were less likely to have epileptic seizures than those given a placebo.^34^ Our study also suggested that depression impacts HRQOL among the patients with epilepsy. Therefore, effective treatment of comorbid depression may benefit quality of life.^35^ Nevertheless, illness‐focused screening alone, such as depression screening, may be inadequate to evaluate patient needs. For example, insomnia is associated with short‐term poor seizure control and worse HRQOL^36^ functioning. Manic/hypomanic symptoms are also associated with poor HRQOL in patients with epilepsy.^37^ In addition, anxiety, independent of depression, is correlated with low HRQOL in patients with epilepsy.^38^ The dose–response relationship between alcohol consumption and epilepsy and unprovoked seizures has previously been reported.^39^ Attention deficit hyperactive disorder (ADHD) also reduces HRQOL in patients with epilepsy.^40^ Unfortunately, it is unreasonable and unrealistic to perform multi‐illness screenings that may affect HRQOL.

Further, traditional screening may miss patients with complex needs who require care coordination. In a Canadian Community Health survey, psychological and social factors impact more on quality of life compared with the biological‐biomedical factor, that is, seizures and their treatment, among 1720 patients with epilepsy.^41^ Seizure frequency has been shown as a strong predictor of HRQOL in patients with epilepsy.^42^, ^43^, ^44^, ^45^ However, in several longitudinal studies, reduced seizure frequency would not improve HRQOL unless patients achieve complete seizure freedom.^46^, ^47^, ^48^ Many studies have consistently shown that psychological factors have a greater degree of association with HRQOL than seizure frequency in individuals who are not seizure free.^9^, ^17^, ^49^, ^50^, ^51^ Our result that seizure frequency was unrelated to HRQOL supports data from these prior investigations.

In other chronic medical conditions, INTERMED complexity scores have been found to be germane in facilitating effective treatment. For example, in patients with multiple sclerosis,^52^ information from the psychological and social domains of INTERMED has promoted amelioration of specific problems that complicate health‐care delivery. Among patients with rheumatoid arthritis, Koch et al.^53^ reported that in comparison to non‐complex patients, individuals with elevated complexity scores on the INTERMED also displayed greater arthritis‐related disabilities and resultant increased health‐care utilization. Our results also indicated that scores on the social and psychological domains of the INTERMED predicted patients HRQOL findings more accurately than data from the biological domain. Non‐clinical factors, such as social and health‐related variables, contribute to treatment resistance and persistent poor health. These are less often assessed during standard evaluations.

The Institute of Medicine’s report^54^ has emphasized the importance of patient centeredness, co‐management for patients with comorbid conditions whose care may cross specialty boundaries, together with coordination of activities involving a team of professionals across disciplines and sectors. To achieve patient‐centered care for all individuals with epilepsy, comprehensive assessments such as complexity assessments including the INTERMED may be essential for coordination of care. In clinical settings, use of the INTERMED would allow care managers to identify patients with increased bio‐psycho‐social‐health system needs. Using the INTERMED data profiles, treatment teams can direct care and organize resources to improve quality of life.

## 5 CONCLUSIONS

The current data suggest the importance of future applied studies involving patients with epilepsy, using the INTERMED complexity instrument. These investigations should be designed to identify areas of complexity that may adversely affect the patient’s HRQOL and when warranted, provide interventions with the goal of improving overall well‐being among patients with epilepsy. If our current data generalize to such future endeavors, procedures such as social skills training may be germane in this regard.

## AUTHOR CONTRIBUTION

YK, IT, HY, TI, and ST contributed to the conception and design of the study; YK, IT, TI, and ST contributed to the acquisition and analysis of data; YK and ST performed statistical analyses; YK, IT, HY, TI, and ST contributed to the first draft. All authors contributed to the final version of the manuscript.

## CONFLICT OF INTEREST

YK has received honoraria for GlaxoSmithKline, MSD, Otsuka, Shionogi, Takeda, Dainippon‐Sumitomo、Meiji, Janssen, and Eisai and has served as a consultant for MSD. IT has received honoraria for GlaxoSmithKline, Pfizer, UCB, Otsuka, Eisai, and Daiichi‐sankyo. None of the other authors has any conflict of interest to disclose.

## ETHICAL APPROVAL

We confirm that we have read the Journal’s position on issues involved in ethical publication and affirm that this report is consistent with those guidelines.
